# Femoral neck system with an additional cannulated screw for the treatment of elderly femoral neck fractures: a retrospective study

**DOI:** 10.3389/fbioe.2025.1688008

**Published:** 2025-12-17

**Authors:** Xiang Yu, Jun Zhang, Yu-Zhi Li, Xiao-Kai Li, Fan-Cheng Chen, Hong-Kui Hu, Xu Li, Bing-Li Liu, Bao-Qing Yu, Rong-Guang Ao

**Affiliations:** Department of Orthopedics, Shanghai Seventh People’s Hospital, Shanghai, China

**Keywords:** femoral neck fracture, femoral neck system, cannulated compression screw, elderly patients, femoral neck shortening

## Abstract

**Objective:**

To compare the clinical efficacy of the femoral neck system (FNS) alone versus FNS combined with an additional cannulated compression screw (CCS) for treating elderly patients (aged 65–75) with Garden I–III femoral neck fractures.

**Methods:**

This retrospective study analyzed 126 patients treated between January 2020 and December 2022. Patients were divided into an FNS group (n = 60) and an FNS+CCS group (n = 66). Key outcomes included operative time, intraoperative blood loss, fluoroscopy frequency, fracture healing time, femoral neck shortening, complications, VAS pain scores, and Harris hip scores at final follow-up.

**Results:**

The two groups showed no significant differences in baseline characteristics. While intraoperative blood loss was comparable, the FNS+CCS group had significantly longer operative times and required more fluoroscopies. All patients were followed for an average of 14.1 months. The FNS+CCS group demonstrated a significantly shorter fracture healing time and a lower incidence of femoral neck shortening at final follow-up. Although VAS scores were similar between groups, the FNS+CCS group achieved significantly higher Harris hip scores. The incidence of complications did not differ significantly.

**Conclusion:**

For elderly patients with femoral neck fractures, FNS fixation augmented with a CCS offers significant advantages over FNS alone, including accelerated fracture healing and superior functional recovery of the hip joint, despite a longer operative time.

## Introduction

Femoral neck fractures represent a prevalent type of fracture among the elderly, constituting 48%–54% of hip fractures. These fractures are frequently managed through joint replacement due to their elevated nonunion rates (Florschutz et al., 2015; O'Connor and Switzer, 2022). However, recent years have witnessed a growing body of literature documenting the efficacy of the Femoral Neck System (FNS) for internal fixation in elderly patients with femoral neck fractures, yielding promising results (Vazquez et al., 2021; Nibe et al., 2022). In comparison to joint replacement, FNS surgery is characterized by its minimally invasive nature, resulting in reduced blood loss, shorter operative duration, and fewer complications (Xu et al., 2023). Consequently, for relatively younger elderly patients (aged 65–75) and those presenting with non-displaced or minimally displaced fractures (Garden types I, II, III), FNS treatment may represent a superior alternative to joint replacement.

Elderly patients frequently present with osteoporosis and reduced bone mass in the femoral neck, which may compromise the holding power of the FNS main screw, potentially leading to complications such as internal fixation loosening, cut-out, and coxa vara (Jiang et al., 2024). To address these challenges, we have implemented a technique in which a cannulated screw (CCS) is inserted parallel to the main nail above the FNS in the coronal plane, thereby enhancing the anti-rotation and anti-varus capabilities of the internal fixation and improving its stability. This study aimed to compare the outcomes of this combined technique with those treated with FNS alone.

## Materials and methods

### Study subjects and design

This study retrospectively analyzed elderly patients aged 65–75 years with femoral neck fractures who underwent internal fixation in the orthopedic department of our hospital between January 2020 and December 2022. Patients who received FNS were categorized into the FNS group, while those who underwent FNS combined with CCS were classified as the FNS+CCS group. All surgeries were performed by the same senior surgeon. Postoperative routine rehabilitation exercises were implemented, and patients were scheduled for regular follow-up that included X-ray examinations. We recorded gender, age, Body Mass Index (BMI), fracture classification, bone density, cause of injury, time from fracture to surgery, operation duration, intraoperative blood loss, number of fluoroscopies, Visual Analog Scale (VAS) score, and Harris hip score at the final follow-up. Additionally, we assessed the quality of fracture reduction, fracture healing time, incidence of internal fixation cut-out, incidence of femoral head necrosis, and femoral neck shortening distance for both patient groups. Before enrollment, subjects were informed about the study protocol, Informed consent was obtained from all patients. All procedures in this study adhered to the ethical principles of clinical research outlined in the Declaration of Helsinki and received approval from the Medical Ethics Committee of Shanghai Seventh People’s Hospital (SSJW-2019085).

### Inclusion and exclusion criteria

Inclusion criteria: (1) age between 65 and 75 years, (2) The fracture type is Garden I, II, and III, (3) presence of a fresh closed femoral neck fracture, (4) normal hip joint function on the injured side prior to injury; Exclusion criteria: (1) presence of Garden IV type fracture, (2) concurrent fractures in other parts of the ipsilateral limb, (3) associated neurovascular injury, (4) involvement of significant organ damage, (5) preexisting abnormal hip joint function, (6) open fracture or pathological fracture.

### Intervention measures

#### Surgical method

General anesthesia is administered, and the patient is positioned supinely on an orthopedic traction table. The healthy lower limb is fixed in a flexed knee and hip position, with external rotation. Under fluoroscopy using a C-arm X-ray machine, traction is applied to the affected limb to achieve reduction of the fracture ends. The traction force and direction are adjusted based on the reduction status to ensure optimal anatomical alignment. In cases where reduction proves challenging, the Kirschner wire ‘seesaw’ technique is employed to facilitate anatomical reduction. Following successful fracture reduction, a 2.5 mm diameter Kirschner wire is inserted anteriorly and superiorly to the femoral neck to temporarily stabilize the fracture ends, thereby preventing loss of reduction during subsequent procedures.

##### FNS group

The C-arm X-ray machine was utilized to conduct anteroposterior and lateral fluoroscopy of the proximal femur on the affected side, followed by the insertion of a central guide pin. The guide pin was accurately positioned at the center of the femoral neck in the anteroposterior view, forming a 130° angle with the femoral lateral locking plate, and was aligned with the central axis of the femoral head and neck in the lateral view. Upon achieving satisfactory fluoroscopic positioning, a 3 cm longitudinal incision was made 2 cm below the greater trochanter, centered on the guide pin. The layers were meticulously dissected to reach the lateral cortex of the femur. A working cannula was inserted, the depth was measured, and the medullary cavity was reamed to accommodate the assembled fixation bolt and plate. Locking screws were subsequently inserted through the plate, with the handle adjusted throughout this process to ensure that the plate was centered along the long axis of the femur. Finally, an anti-rotation screw was driven through the cannula. Further fluoroscopy was conducted, and if separation was observed at the fracture site, a multifunctional rod was employed for compression.

##### FNS+CCS group

**FIGURE 1:**
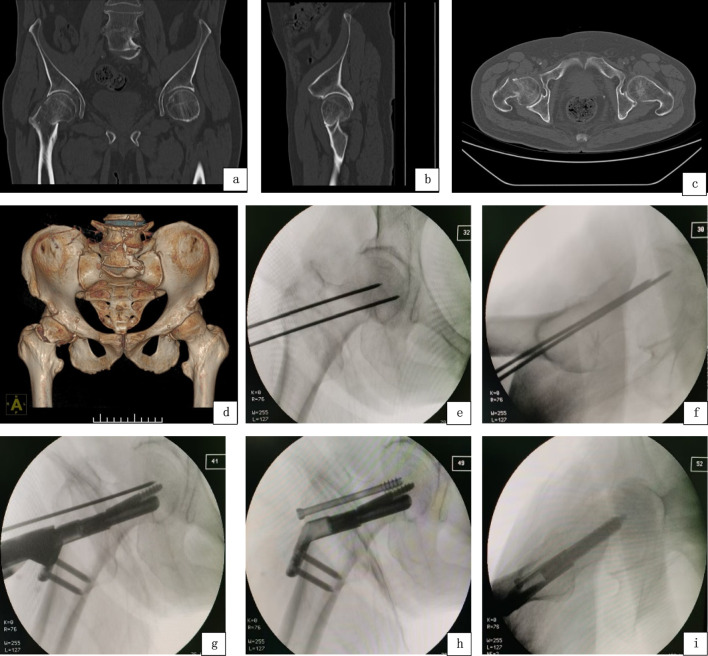
FNS+CCS group, Preoperative CT: **(a)** Coronal plane. **(b)** Sagittal plane. **(c)** Transverse plane. **(d)** Three-dimensional CT; Intraoperative fluoroscopic imaging: **(e,f)** Intraoperative anteroposterior and lateral radiographs showing the placement of the FNS main nail and cannulated screw guide wires; **(g)** Insertion of FNS; **(h)** The hollow screw was inserted, and the internal fixation assembly was in good position; **(i)** Intraoperative lateral fluoroscopy showed good positioning of the internal fixation assembly.

The C-arm X-ray machine was employed to conduct anteroposterior and lateral fluoroscopy of the affected proximal femur. A central guide pin was inserted, positioned in the lower one-third of the femoral neck in the anteroposterior view, forming a 130° angle with the lateral locking plate, and aligned with the central axis of the femoral head and neck in the lateral view. Subsequently, a cannulated screw guide pin was inserted parallel to the central guide pin, located in the upper one-third of the femoral neck in the anteroposterior view and aligned with the central axis of the femoral head and neck in the lateral view. The FNS bolt, anti-rotation screw, and plate were then sequentially implanted and locked. Finally, a 6.5 mm diameter partially threaded cannulated screw (CCS) of appropriate length was implanted above the bolt to enhance the initial stability and rotational strength of the internal fixation, as illustrated in Figure 1.

#### Postoperative treatment

The postoperative management protocols were uniformly applied to both patient groups. Prophylactic antibiotics were administered for a duration of 24 h, oral Celecoxib was prescribed for pain relief, and low molecular weight heparin was utilized to prevent deep vein thrombosis. On the first postoperative day, patients commenced isometric contraction and relaxation exercises of the affected limb muscles, as well as ankle pump exercises. On the second day, patients were instructed to perform non-weight-bearing functional exercises of the affected limb with the aid of a walker, and X-ray imaging was conducted to assess fracture reduction. Follow-up visits were scheduled monthly for the initial 6 months post-discharge, and subsequently every 3 months. Based on the observed degree of fracture healing during follow-up, patients gradually increased their weight-bearing activities until full weight-bearing was achieved.

#### Outcome measures

The following parameters were recorded and assessed: operative time, intraoperative blood loss, and the number of fluoroscopies. To minimize potential bias in the evaluation of subjective outcomes, a blinding procedure was implemented. At the final follow-up, the Harris Hip Score and Visual Analog Scale (VAS) for pain were assessed by an experienced orthopedic surgeon who was not the operating surgeon and was blinded to the patient’s group assignment. Similarly, all radiographic outcomes—including the quality of fracture reduction (Garden index), fracture healing time, femoral neck shortening distance, and the incidence of complications (internal fixation cut-out, nonunion, and femoral head necrosis)—were evaluated by two independent senior radiologists who were blinded to both group allocation and follow-up time points.

Surgical blood loss encompasses both intraoperative and hidden blood loss. The estimation formula for blood loss is as follows: Blood Loss = TBV × (preoperative HCT - postoperative HCT)/preoperative HCT. Here, TBV represents total blood volume, calculated as body weight (kg) multiplied by 70 mL/kg for males or 65 mL/kg for females, while HCT denotes hematocrit (Xu et al., 2021).

The quality of fracture reduction is assessed using the Garden index (Agar and Utkan, 2021), which categorizes fractures into four grades based on radiographic images. Garden Type I: In the anteroposterior view, the angle formed by the central axis of the trabeculae of the femoral head and the medial cortex of the femoral neck ranges from 160° to 180° (normal anatomical angle). In the lateral view, the angle between the axis of the femoral head and the femoral neck is 180° (indicating perfect alignment). The trabecular structures of the femoral head and neck are continuous without any step-off. Garden Type II: In the anteroposterior view, the femoral head exhibits mild valgus (angle <160°), while the trabecular alignment remains essentially continuous. In the lateral view, the angle between the axis of the femoral head and the neck exceeds 150° (indicating mild anteversion or retroversion). Garden Type III: There is significant varus (angle <150°) or valgus (angle >180°) observed in the anteroposterior view, with the angle between the femoral head and neck axis being less than 150° (indicating severe anteversion or retroversion) in the lateral view. Garden Type IV: This type is characterized by complete separation of the femoral head and neck in the anteroposterior view, accompanied by total disruption of the trabecular alignment, and a severe deviation of the femoral head and neck axis (angle <120°) in the lateral view. The interpretation of the X-ray images was independently conducted by two senior radiologists, who performed the classification.

The criteria for assessing fracture healing include the complete disappearance of the fracture line on X-ray images, the absence of longitudinal percussion pain in the affected limb during physical examination, and the absence of significant pain during both active and passive movements. The criterion for identifying implant cut-out is the observation of the implant penetrating the cortex of the femoral head on X-ray images. The criteria for diagnosing femoral head necrosis consist of identifying a focal low-signal area (necrosis focus) within the femoral head on MRI T1WI, as well as a high-signal edema zone (crescent sign) on T2WI. The physical examination was performed by a senior orthopedic surgeon, while the imaging interpretation was conducted independently by two senior radiologists, with the final assessment made by integrating the results from both evaluations.

**FIGURE 2:**
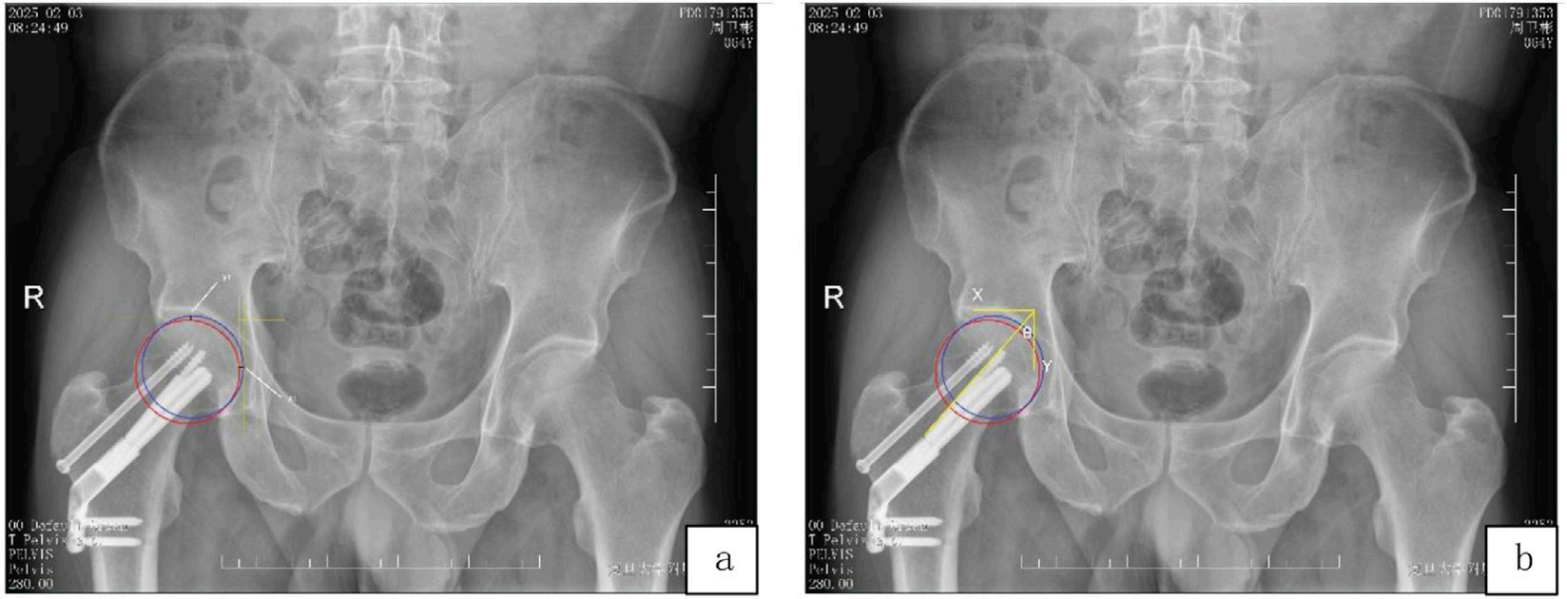
Measurement of femoral neck shortening length after FNS fixation for femoral neck fracture: **(a)** In the anteroposterior pelvic radiograph, superimpose the image of the greater trochanter of the healthy side with that of the affected side. Subsequently, draw a blue circle to represent the femoral head of the healthy side and a red circle for the femoral head of the affected side. The variables x1 and y1 denote the displacement lengths of the femoral head of the affected side relative to the femoral head of the healthy side along the horizontal axis (x-axis) and vertical axis (y-axis), respectively; **(b)** The angle θ represents the angle between the femoral head-neck axis and the vertical axis (y-axis). The femoral neck shortening length |z| (mm) is calculated as |y1 sinθ + x1 cosθ|.

The femoral neck shortening distance was measured using the method proposed by Zlowodzki et al. (2008). Pelvic anteroposterior radiographs obtained at the final follow-up were processed with image processing software Photoshop 2021 (Adobe Inc., United States) to align the images of the greater trochanter on the healthy and affected sides. Concentric circles were drawn with the centers of the femoral heads on both sides until they intersected with the subchondral bone of the femoral head. Horizontal and vertical tangent lines were constructed at the superior and medial edges of these two concentric circles, respectively. The distances between the two vertical tangent lines and the two horizontal tangent lines were measured and recorded as the horizontal displacement (x1) and vertical displacement (y1) of the femoral head. The data were adjusted using the actual diameter of the FNS dynamic rod (10.0 mm), and the angle θ was defined as the angle between the femoral head-neck axis and the vertical y-axis. The vector sum (z) of the horizontal displacement (x1) and vertical displacement (y1) of the femoral head represents the femoral neck shortening length. Based on the value of z, the femoral neck shortening length is categorized into three grades: mild shortening (z ≤ 5 mm), moderate shortening (5 mm < z < 10 mm), and severe shortening (z ≥ 10 mm), as illustrated in Figure 2.

#### Sample size consideration

As this was a retrospective study, an *a priori* power analysis was not conducted prior to patient enrollment. However, a *post hoc* power analysis was performed using G*Power software (Version 3.1.9.7) to assess the statistical power of the primary outcome, which was the Harris Hip Score at the final follow-up. Based on the observed difference between groups (FNS: 85 ± 6 vs. FNS+CCS: 90 ± 5.5), the effect size (Cohen’s d) was calculated to be 0.87, indicating a large effect. With a sample size of 126 patients (60 in FNS group, 66 in FNS+CCS group) and an alpha level of 0.05, the achieved statistical power for the Harris Hip Score exceeded 99%, suggesting that the study had sufficient power to detect this clinically significant difference.

### Statistical analysis

Statistical analysis was performed using SPSS version 24.0. Data are presented as mean ± standard deviation. For quantitative data, analysis of variance or the Wilcoxon test was utilized, depending on the distribution of the data. The Cochran-Mantel-Haenszel (CMH) chi-square test was applied for ordered categorical data, while the Pearson chi-square test or Fisher’s exact probability method was employed for two-category or unordered categorical data. A p-value of less than 0.05 was considered statistically significant.

## Results

### Baseline patient profiles

According to the established inclusion and exclusion criteria, a total of 162 patients aged 65–75 with Garden I, II, and III femoral neck fractures were enrolled in the study. After excluding 25 patients who did not adhere to the follow-up requirements and 11 patients with incomplete imaging data, data from 126 patients were ultimately collected. Among these, 60 patients received FNS treatment, while 66 patients underwent FNS combined with CCS treatment. The baseline characteristics of the two groups—including gender, age, BMI, bone mineral density, cause of injury, time from fracture to surgery, side of fracture, and classification (Garden classification and Pauwels classification)—were compared, revealing no statistically significant differences (p > 0.05). Refer to Table 1 for details.

**TABLE 1:** Comparison of baseline data between the two groups.

Variable	FNS	FNS+CCS	Statistical value	*p* value
Cases	60	66		
Age (years)	68.5 ± 2.7	69.3 ± 2.9	t = −1.60	0.112
Sex (female/male)	34/26	38/28	χ^2^ = 0.106	0.745
BMI(kg/m^2^)	22.6 ± 1.6	22.2 ± 1.4	t = 1.49	0.140
BMD T-score	−1.8 ± 0.5	−1.7 ± 0.6	t = −1.02	0.310
Mechanism of injury				
Fall	52	55	χ^2^ = 0.54	0.461
Car accident	3	5	χ^2^ = 0.35	0.554
Fall from a height	5	6	χ^2^ = 0.014	0.906
Involved side (left/right)	32/28	34/32	χ^2^ = 0.980	0.322
Time from fracture to surgery (x ± s.d.)	3.5 ± 1.5	3.4 ± 1.4	t = −0.386	0.700
Garden classification				
I	2	2	χ^2^ = 0.236	0.889
II	17	16		
III	41	48		
Pauwels classification				
I	3	4	χ^2^ = 0.780	0.680
II	37	44		
III	20	18		

BMI, body mass index; BMD, bone mineral density.

### Clinical efficacy

**FIGURE 3:**
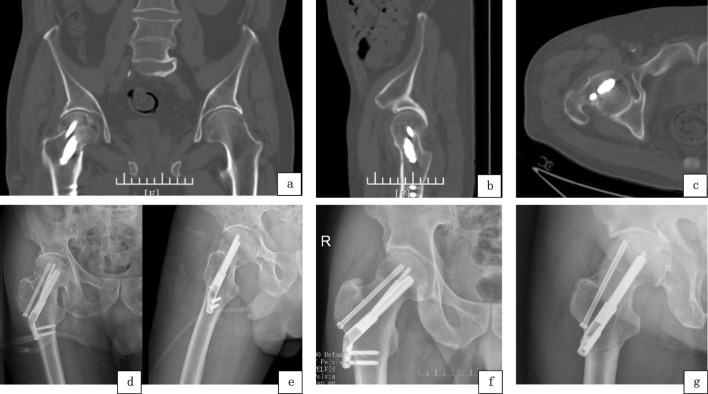
FNS+CCS Group, CT scan 3 days post-operation: **(a)** Coronal plane. **(b)** Sagittal plane. **(c)** Transverse plane; **(d,e)** Postoperative 1-month anteroposterior and lateral X-rays; **(f,g)** One-year postoperative anteroposterior and lateral X-rays.

The incisions in both patient groups healed primarily without complications, including vascular or nerve injuries. There was no statistically significant difference in intraoperative blood loss between the two groups (p > 0.05). However, the FNS+CCS group demonstrated a statistically significant increase in both operative time and the number of fluoroscopies compared to the FNS group (p < 0.01). Postoperative and follow-up imaging is shown in Figure 3.

The follow-up period for both patient groups ranged from 12 to 18 months, with a mean duration of 14.1 months. At the final follow-up, no statistically significant difference was observed in the VAS scores between the two groups (p > 0.05). However, the Harris score for the FNS+CCS group was significantly superior to that of the FNS group, with a statistically significant difference (p < 0.01). Imaging studies indicated no statistically significant difference in the quality of fracture reduction between the two groups (p > 0.05). Nevertheless, the fracture healing time was significantly shorter in the FNS+CCS group, exhibiting a statistically significant difference (p < 0.01). At the final follow-up, the incidence of femoral neck shortening was lower in the FNS+CCS group compared to the FNS group, with a statistically significant difference (p < 0.05). Specifically, regarding the number of cases with shortening, the FNS group exhibited a higher number of cases with mild displacement, which was statistically significant (p < 0.05). In contrast, no statistically significant difference was found in the number of cases with moderate and severe displacement between the two groups (p > 0.05). Additionally, there was no statistically significant difference in the incidence of complications such as nonunion, internal fixation cut-out, and femoral head necrosis between the two groups (p > 0.05). Patients who experienced these complications were subsequently re-admitted for hip arthroplasty. Refer to Table 2.

**TABLE 2:** Comparison of outcome indicators between the two groups.

Outcome indicator	FNS	FNS+CCS	Effect value (95% CI)	*p* value
Cases	60	66		
Operation time (x ± s, min)	48.5 ± 6.6	59.2 ± 6.1	MD = −10.7 (−12.94, −8.46)	<0.01*
Blood loss (x ± s, mL)	84.1 ± 11.0	86.5 ± 9.2	MD = −2.4 (−5.96, 1.16)	0.186
Number of fluoroscopies (x ± s, n)	24.4 ± 4.4	28.6 ± 3.8	MD = −4.2 (−5.56, −2.75)	<0.01*
VAS score (x ± s)	1.8 ± 1.0	1.7 ± 1.0	MD = −0.10 (−0.26, 0.46)	0.577
Harris score (x ± s)	85.0 ± 2.5	90.4 ± 3.2	MD = −5.40 (−6.42, −4.38)	<0.01*
Quality of fracture reduction (n)				
Garden I	51	60	OR = 0.57 (0.19, 1.71)	0.640
Garden II	9	6	OR = 1.77 (0.59, 5.31)	0.305
Fracture healing time (x ± s, m)	4.3 ± 0.7	3.8 ± 0.6	MD = 0.5 (0.26, 0.74)	<0.01*
Femoral neck shortening (n, %)	25 (41.7)	9 (13.6)	OR = 4.52 (1.90, 10.80)	<0.01*
Degree of shortening (n)				
Mild	14	6	OR = 3.04 (1.09, 8.54)	0.048*
Moderate	7	3	OR = 2.77 (0.68, 11.24)	0.200
Severe	4	0	—	0.071
Complications	5 (8.3)	3 (4.5)	OR = 1.91 (0.44, 3.39)	0.350
Nonunion of fracture (n, %)	1 (1.7)	1 (1.5)	OR = 1.10 (0.38, 3.17)	1.000
Internal fixation cut-out (n, %)	1 (1.7)	0 (0)	—	0.499
Femoral head necrosis (n, %)	3 (5)	2 (3.0)	OR = 1.68 (0.13, 3.24)	0.540

MD, mean difference; OR, odds ratio; CI, confidence interval.

*The difference between the two groups was statistically significant.

## Discussion

Femoral neck fractures represent approximately 50% of hip fractures and are associated with substantial disability, mortality, and socioeconomic burden (Stassen et al., 2022). The treatment plan is determined by the patient’s overall health and the degree of fracture displacement (Hatano et al., 2024). For patients under 65 years of age, internal fixation is generally preferred, with options such as three cannulated screws or FNS. Conversely, for patients over 65 years, joint replacement is typically the treatment of choice (O'Connor and Switzer, 2022). However, recent literature has increasingly reported the use of FNS for treating femoral neck fractures in patients over 65 years, with favorable outcomes. Vazquez et al. (2021) documented the use of FNS in treating non-displaced femoral neck fractures in elderly patients over 75 years old, finding that FNS resulted in shorter surgical times and provided greater advantages in preventing secondary fracture displacement. Nibe et al. (2022) treated 25 cases of femoral neck fractures in patients over 65 years with FNS, following up for 5–13 months, and observed that all cases ultimately healed.

This study focused on patients aged 65–75 years with non- or minimally displaced femoral neck fractures (Garden I–III), as their better physiological reserve and higher functional demands make them suitable candidates for joint-preserving surgery. However, osteoporosis in this population can compromise screw fixation stability, increasing risks of implant loosening or cut-out (Mallon et al., 2025). To enhance construct stability, we supplemented the FNS with an additional cannulated screw placed parallel to the main implant. While this added step prolonged operative time and fluoroscopy exposure, the minimally invasive technique resulted in comparable blood loss between groups.

Due to delayed fracture healing and compromised screw fixation from osteoporosis in elderly patients (Hernández-Naranjo et al., 2024), we advised crutch-assisted mobility postoperatively, with weight-bearing gradually increased based on radiographic evidence of healing. This strategy mitigates the risk of postoperative fracture re-displacement. Furthermore, achieving anatomical reduction is critical, as suboptimal reduction increases mechanical stress and may impair vascular supply, elevating the risk of femoral head necrosis (Wang et al., 2019). At the final follow-up, reduction quality assessed by the Garden index (Agar and Utkan, 2021) was excellent in both groups, with most cases classified as Garden type I (FNS group: 51 type I, 9 type II; FNS+CCS group: 60 type I, 6 type II), indicating comparably high-quality reduction in each cohort.

Femoral neck shortening, a common complication after internal fixation, results from periarticular soft tissue traction and fracture site resorption (Zheng et al., 2024). While mild shortening may enhance bone contact, moderate to severe shortening has been shown to impede healing and increase nonunion risk (Wang et al., 2023; Hirakawa et al., 2017). In our study, the FNS+CCS group exhibited significantly reduced shortening incidence and faster healing compared to the FNS group, indicating that the combined construct provides superior stability against rotational and shear forces. Furthermore, shortening compromises hip function by reducing the abductor lever arm, leading to gait asymmetry and poorer outcomes (Polat et al., 2021), which aligns with the significantly higher Harris scores observed in the FNS+CCS group.

This study found that the fracture healing time was shorter in the FNS+CCS group, which is attributed to the mechanical advantages offered by FNS+CCS (Zhu et al., 2025). The addition of a CCS to the FNS improves rotational control and mitigates varus collapse—a particular risk in fractures with posteromedial comminution—by restoring medial support and maintaining reduction (Lopes-Coutinho et al., 2020). Furthermore, this combined fixation method exhibits superior stress conduction, allowing shear forces to be more efficiently converted into compressive stresses, thereby offering excellent dispersion and resistance to shear forces, which promotes optimal fracture healing.

The main complications following internal fixation of femoral neck fractures include nonunion, implant cut-out, and avascular necrosis of the femoral head (Jiang et al., 2024; Davidson et al., 2022). Karl Stoffel (Stoffel et al., 2023) reported a complication rate of 6.4% at 3 months and 8.8% at 12 months in 125 cases of femoral neck fractures treated with FNS. Although the FNS group exhibited a higher number of complications (8.4% vs. 4.5%), the difference was not statistically significant. This may be due to exclusion of Garden IV fractures, a conservative rehabilitation protocol delaying weight-bearing until healing, and a limited mean follow-up of 14.1 months, which may be insufficient to detect all late-onset avascular necrosis cases.

Currently, there is a paucity of research reporting on the outcomes of FNS combined with CCS in the treatment of elderly patients with femoral neck fractures. Bonnaire and Weber (2002) previously compared four methods for fixing unstable Pauwels type III femoral neck fractures: cannulated screws, dynamic hip screws (DHS), DHS combined with anti-rotation screws, and 130° angled plates. The findings indicated that the combination of DHS and anti-rotation screws provided the greatest stability. This study was inspired by Bonnaire’s work and produced similar results, demonstrating that the “plate and screw” fixation method offers superior surgical efficacy.

Despite the promising outcomes, the FNS+CCS technique presents specific challenges. The procedure is more technically demanding, leading to longer operative times and greater fluoroscopic exposure, as evidenced in our results. The technique requires a precise surgical technique to avoid iatrogenic complications and to ensure optimal screw positioning. Therefore, surgeons should be aware of the steeper learning curve associated with this method.

This study has several limitations that should be considered. First, the retrospective, non-randomized design conducted at a single center may introduce potential selection bias, and the fact that all surgeries were performed by a single surgeon limits the generalizability of our findings. Although baseline characteristics were comparable, unmeasured confounding factors could still be present. Second, the mean follow-up period of 14.1 months, while adequate for assessing bone healing, is relatively short for reliably evaluating long-term outcomes such as avascular necrosis of the femoral head. Consequently, the true incidence of this complication might be underestimated. Third, our functional assessment depended entirely on the clinician-reported Harris Hip Score. The absence of patient-reported outcome measures (PROMs) means our study lacks insight into the patients’ own perspectives on their pain, function, and quality of life. Finally, the proposed biomechanical advantages of the combined FNS and CCS technique warrant further investigation through dedicated biomechanical studies.

## Conclusion

In conclusion, augmenting the FNS with an additional CCS provides significant clinical benefits over standalone FNS fixation, including faster fracture healing and improved functional outcomes in elderly patients. This combined approach represents a valuable hip-preserving option, and its promising results warrant further investigation through prospective randomized trials and biomechanical studies.

## Data Availability

The raw data supporting the conclusions of this article will be made available by the authors, without undue reservation.

## References

[B1] Agar A. Utkan A. (2021). The effect of anatomical reduction on functional outcomes in femoral neck fracture: a novel modified garden index. Cureus 13 (11), e19863. 10.7759/cureus.19863 34976490 PMC8712223

[B2] Bonnaire F. A. Weber A. T. (2002). Analysis of fracture gap changes, dynamic and static stability of different osteosynthetic procedures in the femoral neck. Injury 33 (Suppl. 3), C24–C32. 10.1016/s0020-1383(02)00328-5 12423588

[B3] Davidson A. Blum S. Harats E. Kachko E. Essa A. Efraty R. (2022). Neck of femur fractures treated with the femoral neck system: outcomes of one hundred and two patients and literature review. Int. Orthop. 46 (9), 2105–2115. 10.1007/s00264-022-05414-0 35538322 PMC9372123

[B4] Florschutz A. V. Langford J. R. Haidukewych G. J. Koval K. J. (2015). Femoral neck fractures: current management. J. Orthopaedic Trauma 29 (3), 121–129. 10.1097/BOT.0000000000000291 25635363

[B5] Hatano M. Sasabuchi Y. Isogai T. Ishikura H. Tanaka T. Tanaka S. (2024). Increased early complications after total hip arthroplasty compared with hemiarthroplasty in older adults with a femoral neck fracture. Bone Jt. J. 106-B (9), 986–993. 10.1302/0301-620X.106B9.BJJ-2024-0089.R1 39216845

[B6] Hernández-Naranjo J. M. Campuzano-Bitterling B. Renau-Cerrillo M. Vives-Barquiel M. Camacho-Carrasco M. P. Muñoz-Mahamud E. (2024). Preliminary clinical and radiological evaluation of osteosynthesis using the femoral neck system (FNS) for subcapital fractures of the femur. Sci. Reports 14 (1), 14494. 10.1038/s41598-024-64955-z 38914658 PMC11196705

[B7] Hirakawa Y. Nakamura H. Minamitani K. Hashida R. Gotoh M. Shiba N. (2017). Prognostic value of the sliding length of cephalocervical screws to predict the risk of non-union after osteosynthesis: a retrospective analysis of 86 patients with intracapsular femoral neck fractures. J. Orthop. Surg. Res. 12 (1), 33. 10.1186/s13018-017-0533-z 28228129 PMC5322601

[B8] Jiang T. Gao H. Xu B. Lv F. Liu T. (2024). The comparison of femoral neck system and cancellous screws internal fixation for femoral neck fracture. Biotechnol. Genet. Eng. Rev. 40 (3), 1947–1958. 10.1080/02648725.2023.2197335 37043667

[B9] Lopes-Coutinho L. Dias-Carvalho A. Esteves N. Sousa R. (2020). Traditional distance “tip-apex” vs. new calcar referenced “tip-apex” - which one is the best peritrochanteric osteosynthesis failure predictor? Injury 51 (3), 674–677. 10.1016/j.injury.2020.01.024 31983422

[B10] Mallon Z. O. Prentice H. A. Schlauch A. M. Fasig B. H. Paxton E. W. Sadeghi C. (2025). Femoral neck system compared with 3 cannulated screws in the treatment of femoral neck fracture in patients aged 60 and older: a multicenter registry-based study. J. Bone Jt. Surg. Am. 107 (9), 958–967. 10.2106/JBJS.24.00781 40153479

[B11] Nibe Y. Matsumura T. Takahashi T. Kubo T. Matsumoto Y. Takeshita K. (2022). A comparison between the femoral neck system and other implants for elderly patients with femoral neck fracture: a preliminary report of a newly developed implant. J. Orthop. Sci. 27 (4), 876–880. 10.1016/j.jos.2021.04.016 34090779

[B12] O'Connor M. I. Switzer J. A. (2022). AAOS clinical practice guideline summary: management of hip fractures in older adults. J. Am. Acad. Orthop. Surg. 30 (20), e1291–e1296. 10.5435/JAAOS-D-22-00125 36200817

[B13] Polat A. Misir A. Buyukkuscu M. O. Basilgan S. Basar H. (2021). Factors associated with femoral neck shortening after closed or open reduction and screw fixation. Indian J. Orthop. 56 (2), 303–311. 10.1007/s43465-021-00484-5 35140862 PMC8789974

[B14] Stassen R. C. Jeuken R. M. Boonen B. Meesters B. de Loos E. R. van Vugt R. (2022). First clinical results of 1-year follow-up of the femoral neck system for internal fixation of femoral neck fractures. Arch. Orthop. Trauma Surg. 142 (12), 3755–3763. 10.1007/s00402-021-04216-0 34734328

[B15] Stoffel K. Michelitsch C. Arora R. Babst R. Candrian C. Eickhoff A. (2023). Clinical performance of the femoral neck system within 1 year in 125 patients with acute femoral neck fractures, a prospective observational case series. Arch. Orthop. Trauma Surg. 143 (7), 4155–4164. 10.1007/s00402-022-04686-w 36460761 PMC10293436

[B16] Vazquez O. Gamulin A. Hannouche D. Belaieff W. (2021). Osteosynthesis of non-displaced femoral neck fractures in the elderly population using the femoral neck system (FNS): short-term clinical and radiological outcomes. J. Orthop. Surg. Res. 16 (1), 477. 10.1186/s13018-021-02622-z 34348753 PMC8336369

[B17] Wang Y. Ma J. X. Yin T. Han Z. Cui S. S. Liu Z. P. (2019). Correlation between reduction quality of femoral neck fracture and femoral head necrosis based on biomechanics. Orthop. Surg. 11 (2), 318–324. 10.1111/os.12458 31025811 PMC6594541

[B18] Wang K. Lin D. Chen P. Lin C. Feng T. Liu J. (2023). Incidence and factors influencing neck shortening after screw fixation of femoral neck fractures with the femoral neck system. J. Orthop. Surg. Res. 18 (1), 317. 10.1186/s13018-023-03787-5 37095563 PMC10127061

[B19] Xu X. Zhu Q. Yang Y. Wang L. Chen X. (2021). Investigation of perioperative blood loss of femoral shaft fractures treated with intramedullary nail or locking compression plate. Injury 52 (7), 1891–1896. 10.1016/j.injury.2021.04.018 33853738

[B20] Xu X. Fan J. Zhou F. Lv Y. Tian Y. Ji H. (2023). Comparison of femoral neck system to multiple cancellous screws and dynamic hip screws in the treatment of femoral neck fractures. Injury 54 (Suppl. 2), S28–S35. 10.1016/j.injury.2022.03.041 35367076

[B21] Zheng S. Lin D. Chen P. Lin C. Chen B. Zheng K. (2024). Comparison of femoral neck shortening after femoral neck system and cannulated cancellous screw fixation for displaced femoral neck fractures in young adults. Injury 55 (6), 111564. 10.1016/j.injury.2024.111564 38640596

[B22] Zhu Y. Wu S. Yan J. Wang W. Huang X. Zhang H. (2025). Finite element analysis of the Femoral neck system for different placement positions in the fixation of Pauwels type Ⅲ femoral neck fractures. Injury 56 (4), 112218. 10.1016/j.injury.2025.112218 40088553

[B23] Zlowodzki M. Brink O. Switzer J. Wingerter S. Woodall J. Petrisor B. A. (2008). The effect of shortening and varus collapse of the femoral neck on function after fixation of intracapsular fracture of the hip: a multi-centre cohort study. J. Bone Jt. Surg. Br. 90 (11), 1487–1494. 10.1302/0301-620X.90B11.20582 18978271

